# Development of an amoxicillin-radix scutellaria extract formulation and evaluation of its pharmacokinetics in pigs

**DOI:** 10.1186/s12917-023-03713-1

**Published:** 2023-09-19

**Authors:** Dandan Yi, Xuemei Wen, Wei Xu, Yangfeng Xu, Xin Deng, Guoqing Yan, Liqin Wu, Qiuling Liang, Zhengmin Liang, Jianbo Peng, Jiakang He

**Affiliations:** 1 College of Animal Science and Technology, Guangxi University, Room 124, 100 Daxue Road, Xixiangtang District, Nanning, Guangxi 530005 P. R. China; 2 Guangxi Key Laboratory of Animal Breeding, Disease Control and Prevention, Nanning, 530004 P. R. China; 3 Guangxi Zhuang Autonomous Region Engineering Research Center of Veterinary Biologics, Nanning, 530004 P. R. China; 4 Department of Animal Science and Technology, Guangxi Agricultural Vocational College, Nanning, 530007 P. R. China

**Keywords:** Amoxicillin, Radix scutellaria extract, Bacteriosis, Pharmacokinetics, Pig

## Abstract

**Background:**

A new antibacterial compound powder of amoxicillin (AMO)/Radix Scutellaria extract (RSE) was developed, and its pharmacokinetics were determined in pigs following oral administration.

**Results:**

The MIC ranges of AMO against *Escherichia coli*, *Staphylococcus aureus* and *Streptococcus* were 1–8 μg/mL, 0.5–4 μg/mL and 0.5–64 μg/mL, respectively. The MIC ranges of RSE against *E. coli*, *S. aureus*, and *Streptococcus* were greater than 2.5 mg/mL, 0.156–2.5 mg/mL, and greater than 2.5 mg/mL, respectively. For *S. aureus*, the combined drug susceptibility test showed that AMO and RSE had an additive or synergistic effect. The results of compatibility test, the excipient screening test and the drug quality control test showed that the formulation had stable quality and uniform properties under the test conditions. Two studies were conducted to investigate the pharmacokinetics of the compound product in pigs. First, the pharmacokinetics of the AMO-RSE powder were compared with those of their respective single products. The results showed no significant change in the main pharmacokinetic parameters when either component was removed from the compound formulation; thus, AMO and RSE have no pharmacokinetic interaction in pigs. Second, pigs were orally administered three different doses of AMO-RSE powder. The Cmax and AUC increased proportionally with increasing p.o. dose; thus, the λ_z_, t_1/2λ_, MRT, and T_max_ were unchanged for the doses of 10, 20, and 30 mg/kg AMO and the doses of 5, 10, and 15 mg/kg BCL, showing that AMO/baicalin in AMO-RSE powder showed linear pharmacokinetic characteristics in pigs.

**Conclusions:**

The combined drug sensitivity test of AMO and RSE against S. aureus showed that the combination was additive or synergistic. Pharmacokinetic studies indicated that AMO and BCL do not interfere with each other in pigs when used in a compound formulation. The pharmacokinetic parameters remained unchanged regardless of the dose for p.o. administration, indicating linear pharmacokinetic properties over the tested dose range. The quality of the AMO-RSE powder was good and stable, providing a foundation for its clinical application in veterinary medicine. Further bioavailability, PK/PD and clinical trials are still needed to determine the final dosage regimen.

## Background

Bacterial infections are the second leading cause of global mortality [1]. These infections not only endanger human life but also cause large economic losses to the agricultural industry, such as piglet acute diarrhea caused by mixed bacterial infection [2]*. Salmonella pullorum* can induce avian salmonellosis after infection in poultry, causing recessive infection and even death [3], and *Staphylococcus aureus* has led to serious mastitis and endometritis in infected dairy cows [4]. Antibiotics play a very important role in the treatment of bacterial infections [5], but the issue of antibiotic resistance should not be neglected. Conventional drugs administered in new ways or prepared as compound formulations might be a strategy to combat antibiotic resistance and increase antibiotic sensitivity [6]. Amoxicillin (AMO), a classic antibiotic, has been in use since the 1970s [7]; this antibiotic is a pharmaceutical widely employed in veterinary medicine worldwide [8], such as for the control of severe, systemic infections such as *Streptococcus* in pigs [9]. However, with the advent of the antibiotic era, the overuse and inappropriate consumption and application of antibiotics have driven the rapid emergence of multidrug-resistant pathogens [10]. In recent years, there has been a common phenomenon that using AMO alone in bacteriosis does not maximize its effectiveness. Therefore, it is of interest to develop a new AMO formulation for the treatment of bacterial infections.

*Scutellaria baicalensis* has been widely used as a medicinal plant in China for thousands of years, and the preparation from its roots is called Huang-Qin. This preparation has been applied in the treatment of diarrhea, cardiovascular diseases, inflammation, and respiratory infections [11–14]. Radix Scutellaria extract (RSE) was obtained from *S. baicalensis*, baicalin (BCL) is the active ingredient in RSE, and the content of BCL in RES is not less than 85% according to the Chinese veterinary pharmacopoeia. It has been reported that BCL has many pharmacological activities, such as antibiosis, anti-inflammatory, antioxidation, antiviral and antitumor activities [15–19]. The antibacterial mechanism of BCL may modulate the oxidative stress response [20], attenuate quorum sensing-controlled virulence [21], modulate both bacterial virulence and host response [22], and other causes. According to a report, BCL has a synergistic antibacterial effect when combined with other antimicrobial drugs. For instance, Novy P determined that BCL acts synergistically with oxytetracycline and tetracycline, enhancing their antimicrobial activity against *S. aureus* [23] in vitro. Leung KC found that *S. baicalensis* and nanoparticle-encapsulated chlorhexidine can enhance synergistic antibacterial effects in common oral bacterial biofilms [24]. Wang J thought BCL may be used as a natural agent resistance inhibitor for azithromycin-resistant *S. saprophyticus*, reducing the development of azithromycin resistance [25].

To maximize the effect of AMO, scientists have tried to prepare AMO in compound formulations to improve its efficacy, and many AMO compound formulations have been studied and applied, such as AMO/clavulanic acid, AMO/cephalosporins, AMO/sulbactam, AMO/clarithromycin, AMO/apramycin, and AMO and cefotaxime [26–31]. Currently, traditional Chinese herbal medicine (TCHM) and its constituents are considered potential sources of new antimicrobial agents [32]. However, there are few reports about compound formulations of AMO and active pharmaceutical ingredients (APIs), especially compound formulations of AMO and BCL.

Therefore, based on the antibacterial effect of AMO and BCL, this study's objective was to prepare AMO-RSE powder by prescription screening and investigate its pharmacokinetics in pigs to provide a basis for the clinical application of compound preparations and to further provide a safe and effective AMO-RSE compound preparation for veterinary use.

## Results

### Results of the antibacterial susceptibility test

As shown in Table 1, the MICs of AMO against *Escherichia coli*, *Salmonella* and *Staphylococcus aureus* were 1–8 μg/mL, 0.25–32 μg/mL and 1-128 μg/mL, respectively. The MICs of RSE against *E. coli*, *Salmonella*, and *S. aureus* were greater than 2.5 mg/mL, 0.3–2.5 mg/mL, and greater than 2.5 mg/mL, respectively. As shown in Table 2, the combined drug sensitivity test of AMO and RSE against *S. aureus* was additive or synergistic.

**Table 1:** Results of the drug sensitivity test of AMO

Bacteria	Numbers	RSE MIC (mg/mL)	AMO MIC (μg/mL)
*E. coli*	15	> 2.5	1–8
*S. aureus*	16	0.3–2.5	0.25–32
*Streptococcus*	16	> 2.5	1–128

**Table 2:** Results of the combined drug sensitivity test of RSE and AMO against *Streptococcus*

Bacteria	Synergy	Additive	Irrelevant	Antagonism
*Streptococcus* (16)	4 (16)	12 (16)	0 (0)	0 (0)

### Preparation of powder—compatibility testing and excipient screening

The compatibility between APIs plays a very important role in preparing a compound powder and further affects the stability of the formulation. The appearance of the AMO-RSE preparation was a yellow powder under the experimental conditions of high temperature, high humidity, and strong light. The contents, properties, APIs and related substances of AMO-RSE powder showed no significant changes after exposure to experimental conditions. This result is indicated that AMO and BCL had good compatibility and could be successfully prepared as compound formulations.

To produce the powder formulation, in this study, five excipients, including glucose, sucrose, corn starch, cassava starch and dextrin, were used to select the best excipient to prepare AMO-RSE compound powder. The results of relative density, relative moisture absorption, and angle of repose showed that glucose was the best excipient; the relative density of the selected excipients was 1.5 ~ 1.6 g/mL, the sucrose moisture absorption percentage of glucose was the lowest, and its angle of repose was 41.44°, which was relatively small compared with that of the four other excipients.

For AMO-RSE powder processing, after passing all components through an ASTM #80 mesh sieve, the APIs AMO and RSE were mixed using a V-shell blender (GlobePharma, Maxiblend®, New Brunswick, NJ, USA); then, the mixtures were combined with glucose, and the ratio of AMO:RSE:glucose was 10:5:85. Quality evaluation experiments were subsequently carried out to determine the quality of the compound formulation.

### Quality inspection of the powder formulation

Through the critical relative moisture absorption and uniformity inspection, the test results show that the relative humidity of AMO-RSE powder was 80.05% under the experimental conditions. The coefficient of variation of both formulations was less than 5%, the difference was not significant, and the properties were uniform. In addition, the contents of AMO and BCL in the powder formulation were determined.

### Pharmacokinetic study

#### Initial pharmacokinetic study

**Fig. 1:**
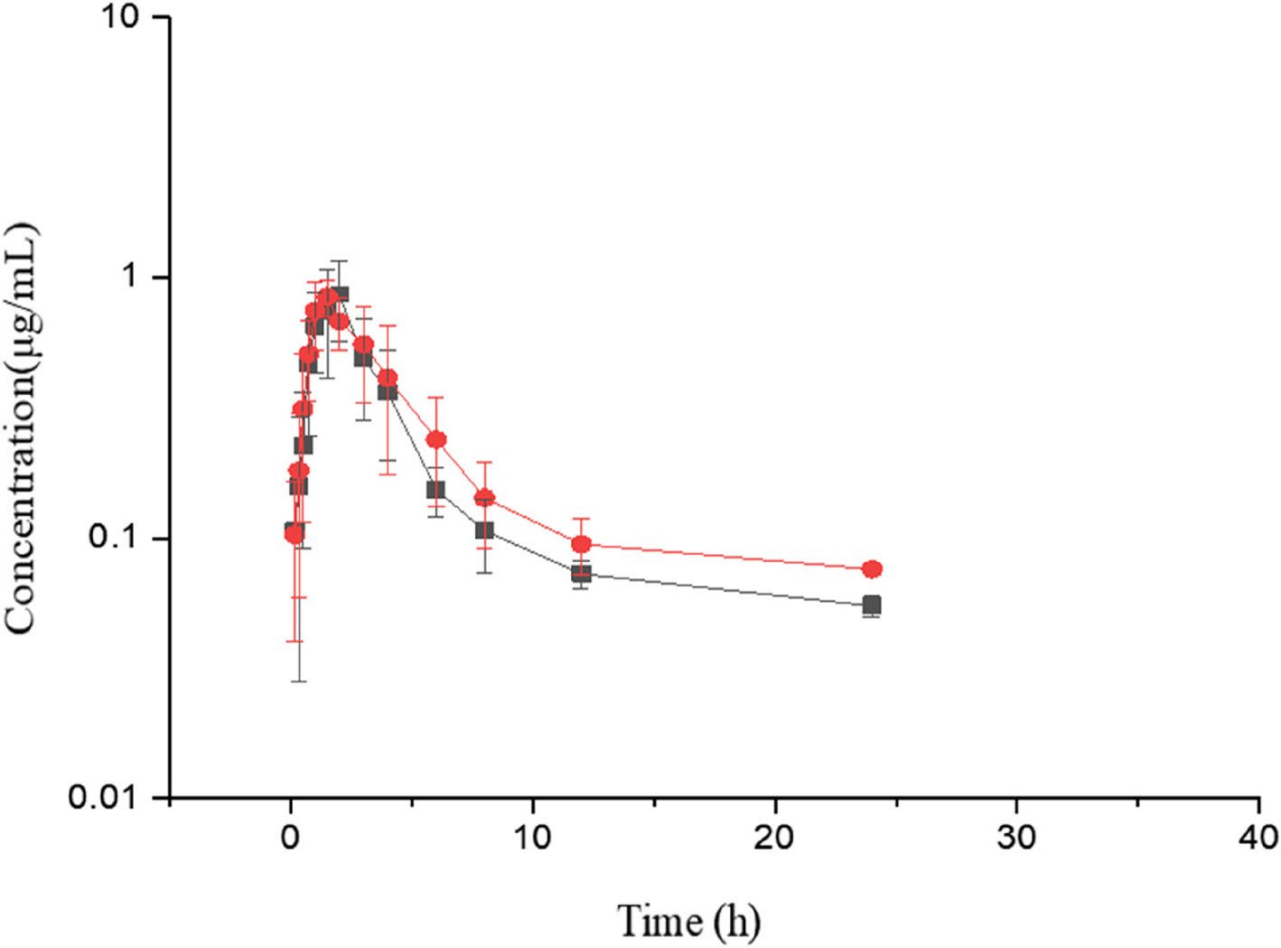
Plasma concentrations (mean ± SD) of amoxicillin (μg/mL) in pigs (*n* = 6) after oral administration at a dose of 10 mg/kg amoxicillin (AMO). ‘‘  ’’ indicates AMO-RSE powder, and ‘‘  ’’ indicates AMO powder without baicalin (BCL)

The plasma AMO concentrations (mean ± SD) at different time points are illustrated in Fig. 1. The pharmacokinetic parameters and the results of statistical analysis are reported in Table 3. The pharmacokinetic data of AMO-RSE powder were not significantly different (*P* > 0.05) from AMO-RSE powder without BCL.

**Table 3:** Comparison of plasma AMO pharmacokinetic parameters after single-dose oral administration of AMO-RSE powder and AMO powder (AMO 10 mg/kg) (*n* = 6)

Parameters	AMO-RSE powder		AMO powder		*P* value
	Mean	SD	Mean	SD	
λ_z_ (1/h)	0.1843	0.1313	0.2087	0.1001	0.726
t_1/2λ_ (h)	6.0402	3.9833	3.9712	1.7612	0.283
AUC_0-∞_ (µg·h/mL)	4.1215	0.9592	4.3558	1.6442	0.771
AUMC_0-∞_ (µg·h^2^/mL)	34.2215	21.1752	27.9650	20.7409	0.616
MRT_0-∞_ (h)	7.9894	4.3668	5.8845	2.0788	0.321
C_max_ (µg/mL)	0.9443	0.2510	0.9205	0.1433	0.845
T_max_ (h)	Median	Range	Median	Range	
	1.5833	1.00 ~ 2.00	1.2500	1.00 ~ 1.50	0.186

**Fig. 2:**
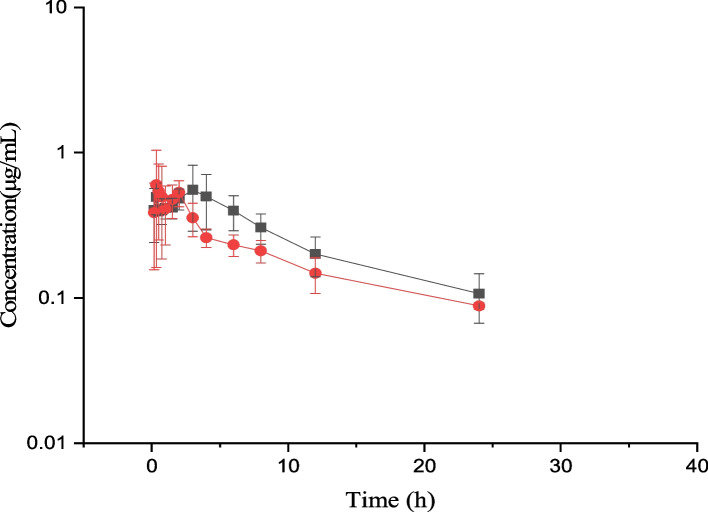
Plasma concentrations (mean ± SD) of BCL (μg/mL) in pigs (*n* = 6) after oral administration at a dose of 10 mg/kg BCL. ‘‘  ’’ indicates AMO-RSE powder, and ‘‘  ’’ indicates BCL powder without AMO

For BCL, the plasma concentrations (mean ± SD) at different times are illustrated in Fig. 2. The pharmacokinetics parameters and the results of statistical analysis can be seen in Table 4. It was shown that the pharmacokinetic parameters of AMO-RSE powder were not significantly different (*P* > 0.05) from AMO-RSE powder without AMO.

**Table 4:** Comparison of plasma BCL pharmacokinetic parameters after single-dose oral administration of AMO-RSE powder and RSE powder (BCL 5 mg/kg) (*n* = 6)

Parameters	AMO-RSE powder		RSE powder		*P* value
	Mean	SD	Mean	SD	
λ_z_ (1/h)	0.110	0.061	0.094	0.061	0.670
t_1/2λ_ (h)	7.615	2.921	9.210	3.758	0.432
AUC_0-∞_ (µg·h/mL)	6.978	1.698	5.357	1.454	0.107
MRT_0-∞_ (h)	11.520	3.775	12.433	5.045	0.731
C_max_ (µg/mL)	0.632	0.222	0.730	0.348	0.575
T_max_ (h)	Median	Range	Median	Range	
	1.833	0.33 ~ 4.00	1.194	0.33 ~ 2.00	0.436

#### Second pharmacokinetic study

**Fig. 3:**
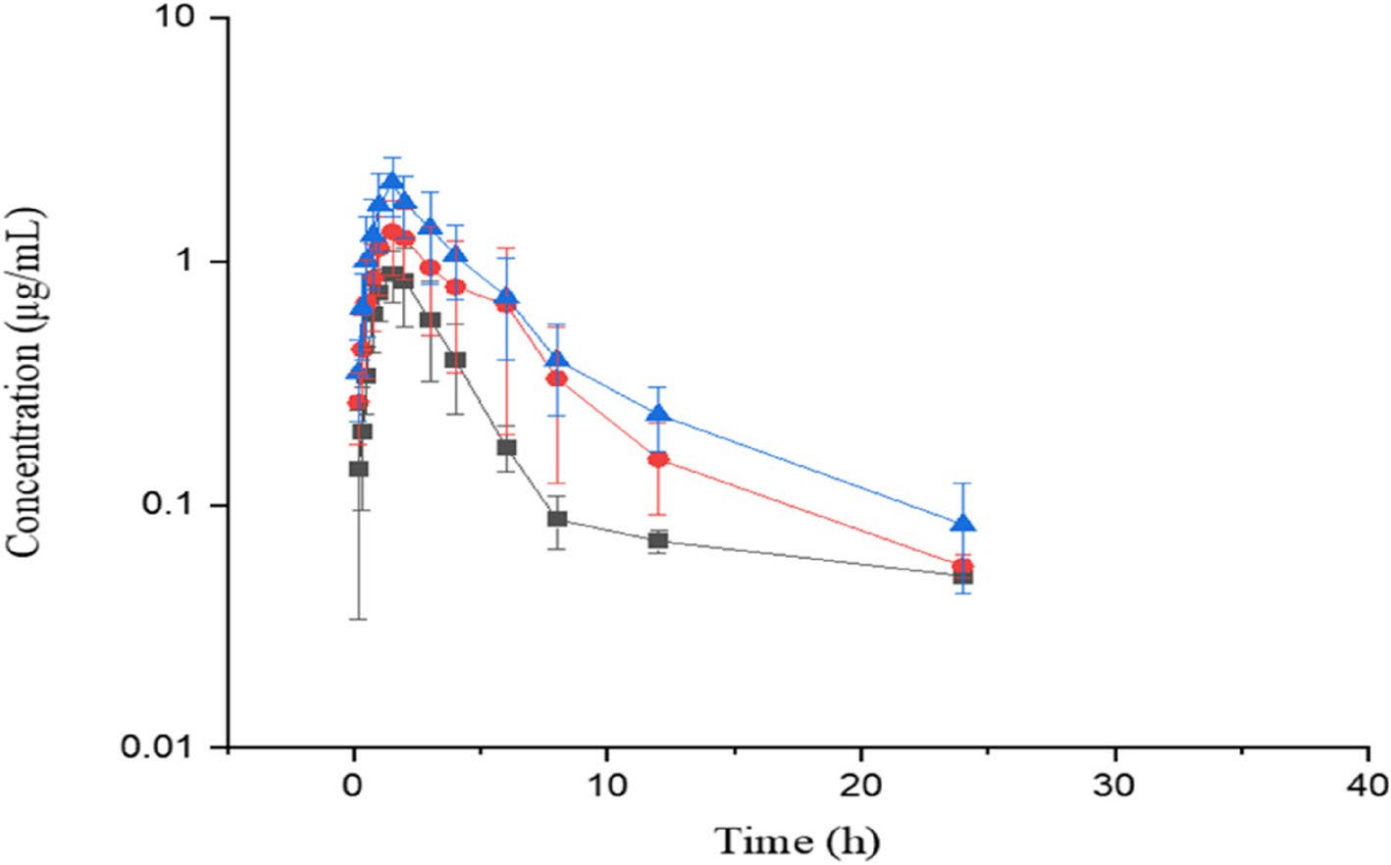
Plasma concentrations (mean ± SD) of AMO (μg/mL) in pigs (*n* = 6) after oral administration at doses of 10, 20 and 30 mg/kg AMO. ‘‘  ’’ indicates10 mg/kg ‘‘  ’’, indicates 20 mg/kg, and ‘‘  ’’ indicates 30 mg/kg AMO-RSE powder administration

Figure 3 shows the mean plasma concentrations of AMO following p.o. AMO-RSE powder administration at three different doses of 10, 20 and 30 mg/kg of AMO. The pharmacokinetic parameters are reported in Table 5. After a single p.o. administration, λ_z_, t_1/2λ_, MRT, and T_max_ were unchanged at the doses of 10, 20, and 30 mg/kg AMO, and C_max_ and AUC increased proportionally with increasing p.o. dose, indicating that AMO exhibited linear pharmacokinetic properties in the range of 10–30 mg/kg.

**Table 5:** Mean (± SD) pharmacokinetic parameters of AMO from study two after p.o. AMO-RSE powder at dosages of 10, 20 and 30 mg/kg AMO in pigs (*n* = 6)

Parameters	10 mg/kg		20 mg/kg		30 mg/kg	
	Mean	SD	Mean	SD	Mean	SD
λz (1/h)	0.18	0.14	0.18	0.15	0.17	0.16
t1/2λ (h)	6.21	3.84	6.45	3.74	6.70	3.77
AUC0-∞ (µg·h/mL)	4.80	1.74	9.37	2.61	12.70	3.13
AUMC0-∞ (µg·h2/mL)	34.41	21.89	58.16	18.84	93.44	45.15
MRT0-∞ (h)	8.03	4.60	8.22	4.34	8.15	4.71
Cmax (µg/mL)	0.91	0.45	1.71	1.57	2.82	1.14
Tmax (h)	Median	Range	Median	Range	Median	Range
	1.65	0.75–2	1.65	1.0–2	1.65	0.75–2

**Fig. 4:**
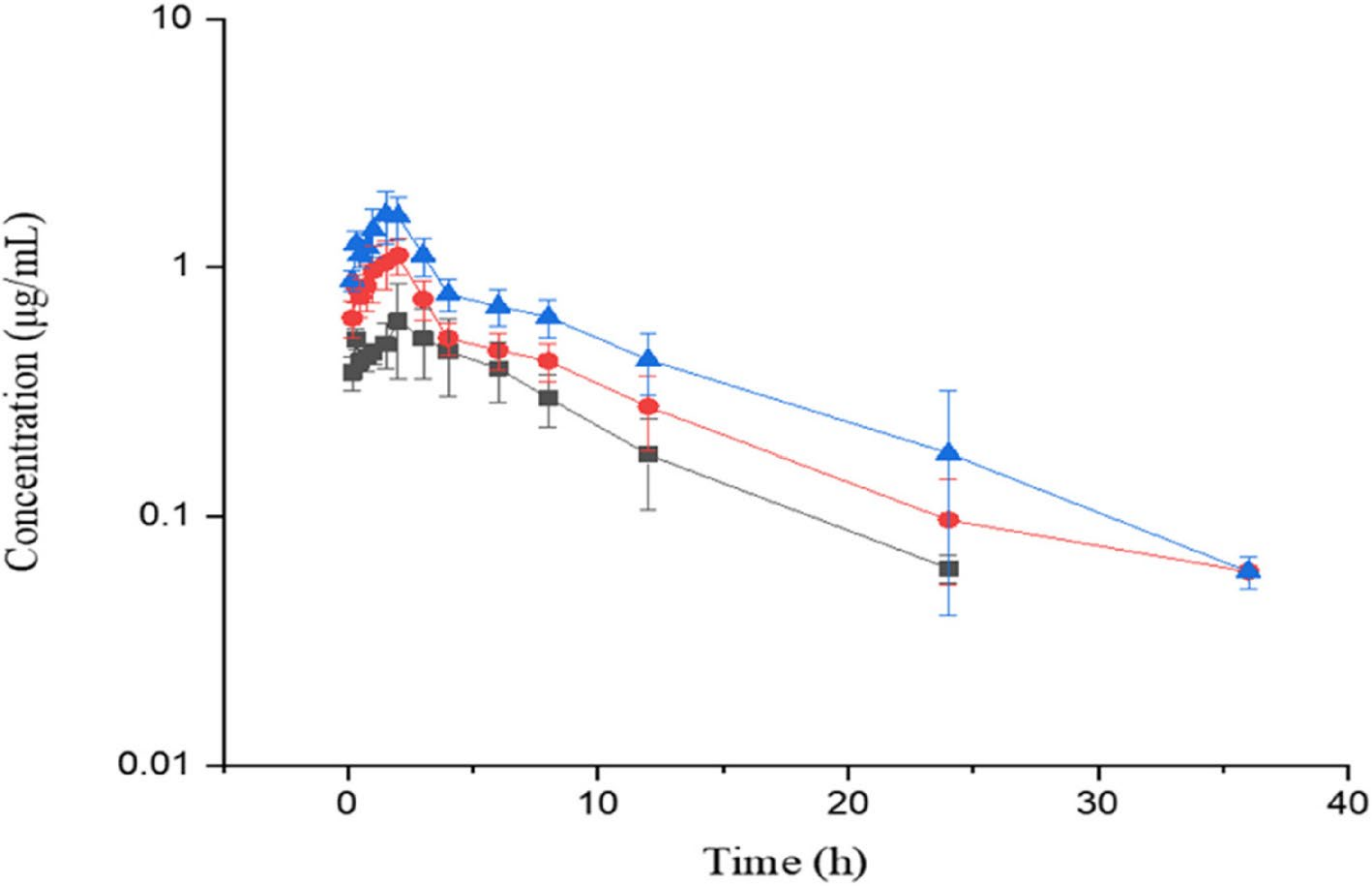
Plasma concentrations (mean ± SD) of BCL (μg/mL) in pigs (*n* = 6) after oral administration at doses of 5, 10 and 15 mg/kg BCL. ‘‘  ’’ indicates 10 mg/kg, ‘‘  ’’ indicates 20 mg/kg, and ‘‘  ’’ indicates 30 mg/kg AMO-RSE powder

The plasma BCL concentrations (mean ± SD) at different time points are illustrated in Fig. 4 following p.o. AMO-RSE powder at three different doses of 5, 10 and 15 mg/kg BCL. The pharmacokinetic parameters are shown in Table 6. Similar to AMO, the Cmax and AUC values of BCL presented dose dependence, indicating that BCL had linear pharmacokinetic properties over the tested dose range.

**Table 6:** Mean (± SD) pharmacokinetic parameters of BCL from study two after p.o. AMO-RSE powder at doses of 5, 10 and 15 mg/kg BCL in pigs (*n* = 6)

Parameters	5 mg/kg		10 mg/kg		15 mg/kg	
	Mean	SD	Mean	SD	Mean	SD
λ_z_ (1/h)	0.14	0.05	0.09	0.01	0.10	0.02
t_1/2λ_ (h)	5.70	1.89	7.65	1.23	7.57	2.00
AUC_0-∞_ (µg·h/mL)	5.96	1.33	9.91	1.94	15.08	3.94
AUMC_0-∞_ (µg·h^2^/mL)	52.95	22.27	102.09	34.84	155.79	72.72
MRT_0-∞_ (h)	8.67	2.23	10.11	1.67	9.92	2.06
C_max_ (µg/mL)	0.64	0.25	1.92	0.66	1.79	0.34
T_max_ (h)	Median	Range	Median	Range	Median	Range
	2.00	0.75–3.00	2.00	1.00–3.00	2.00	1.50–2.00

## Discussion

With increasing bacterial drug resistance in recent years, optimizing and maximizing existing classic antibiotics is an optional strategy for combatting such bacterial drug resistance [33]. The unreasonable use of these antibiotics leads to an increase in bacterial drug resistance. Preparing an AMO compound formulation is a good strategy for decreasing the bacterial drug resistance of AMO[34]. BCL, an active ingredient extracted from *S. baicalensis*, has been used in the Chinese market for years, and the API form of BCL (content > 85%) is RSE. Because of its advantages in terms of price and quality, RSE is of notable interest in the pharmaceutical industry. The use of combinations of BCL and many antibiotics, such as levofloxacin and osthole, has been reported [35, 36]. However, there are few studies on AMO combinations with BCL monomers. Therefore, in this study, we aimed to develop a new AMO-RSE formulation for application to prevent and treat bacterial infection in pigs and reduce bacterial drug resistance development. Group medicinal application is the most commonly used mode of administration in the pig industry, and the dose form of powder is an optional method for group medicinal application. The rationality of a formulation is a key component in preparing a compound formulation. Therefore, this study aimed to conduct drug sensitivity tests and combined drug sensitivity tests to evaluate the prescription rationality of the formulation. Common pathogenic bacteria in the veterinary clinic, *E. coli*, *S. aureus* and *Salmonella*, were selected for single-drug sensitivity tests. The MIC results of AMO and RSE on the test strains are shown in Table 1. As shown in Table 1, RSE can inhibit the growth of *S. aureus*, which is related to its inhibition of biofilm formation [37]. On this basis, a combined drug sensitivity test of both drugs against *S. aureus* was carried out. The results are shown in Table 2. The combination of the two drugs shows a synergistic or additive effect, which provides a scientific basis for the research and development of antibacterial drug formulations and indicates the rationality of the AMO-RSE compound formulation.

Compatibility is very important when preparing compound formulations. If there are physical and chemical reactions between raw materials, they are not suitable for use together in a compound formulation [38]. In this study, under the conditions of high temperature, strong light, and high humidity, the content of the main drug in the AMO-RSE powder was measured. These results show that there was no significant interaction between the two APIs under the conditions of influencing factors. The two drugs could be mixed without impacting the drug content, and this result provides a basis for the reasonable compatibility of AMO and BCL.

To be more conducive to production, transportation, and storage, it is necessary to select optimal auxiliary materials, namely, excipients [39, 40]. The excess moisture absorption rate, relative density, and fluidity of auxiliary materials can be detrimental to the production and storage of formulations under normal conditions. The test results of the moisture absorption rate, relative density, and angle of repose showed that the moisture absorption rate, relative density, and angle of repose of anhydrous glucose were the best among the screened excipients. Therefore, anhydrous glucose was chosen as the excipient to reduce the moisture absorption efficiency of AMO-RSE powder, increase the fluidity between drugs, keep the formulation fully mixed and maintain a uniform state.

The pharmacokinetic interaction between compound preparations is not only a scheme to investigate the rationality of prescriptions but also a foundation for guiding the use of drugs in the clinic. If an interaction of pharmacokinetics occurs in a compound formulation, it might not be suitable for preparing a compound formulation or for adjusting the dose of the formulation. Pharmacokinetic interactions might occur for the properties of absorption, distribution, metabolism or excretion. Therefore, the major pharmacokinetic parameters t1/2 λ, MRT, Tmax, Cmax, AUC and AUMC were used to investigate the differences in the compound formulation and the formulation without AMO or BCL. As shown in Table 3, the t1/2 λ, MRT, Tmax, Cmax, AUC and AUMC of AMO-RSE powder were not significantly different from those of AMO powder without BCL. Similarly, there were no significant differences between the AMO-RSE powder and RSE powder without AMO (Table 4).

The pharmacokinetic behavior of three different doses of AMO-RSE compound powder showed that the Cmax and AUC increased proportionally with increasing p.o. doses. The pharmacokinetic parameters Cmax and AUC of AMO or BCL in AMO-RSE powder have a good linear relationship with the dose (*r* > 0.99), and their values are independent of dose. The results showed that AMO/BCL in AMO-RSE powder showed linear pharmacokinetic characteristics in pigs. Based on the linear pharmacokinetic characteristics and considering the results of synergistic or additive effects on the combined drug susceptibility, the recommended dose of AMO-RSE powder for the treatment of bacterial infections can be adjusted in a range from 10–30 mg/kg AMO and 5–15 mg/kg BCL. Further pharmacokinetic/pharmacodynamic and clinical studies should be performed to confirm the final optimal dose of AMO-RSE powder.

## Conclusion

The combined drug sensitivity test of AMO and RSE against *S. aureus* was additive or synergistic. Pharmacokinetic studies indicated that AMO and BCL do not interfere with each other in pigs when used in a compound formulation. The pharmacokinetic parameters remained unchanged regardless of the dose for p.o. administration, indicating linear pharmacokinetic properties over the tested dose range. The quality of the AMO-RSE powder was good and stable, showing potential in veterinary applications for the prevention and treatment of bacterial infectious diseases.

## Methods

### Test strains

Fifteen strains of *E. coli*, 16 strains of *S. aureus*, and 16 strains of *S. aureus* were provided by the Department of Pharmacology and Toxicology of China Agricultural University.

### Antibacterial susceptibility testing

The minimum inhibitory concentration (MIC) of AMO and RSE was tested in the bacteria mentioned above; this method was previously reported by Jiarong Cao, et al. [41].

Amoxicillin or baicalin was added to a sterile 96-well plate with multiple dilutions from low to high concentrations from the first well to the tenth well, with 100 μL per well. 100 μL of bacterial solution was added to a concentration of 1 × 10^6^ CFU/mL. 200 μL of the bacterial solution was added to the 11th well as positive control, and 200 μL of broth was added to the 12th well as a negative control. The MIC test of quality control bacteria was carried out at the same time in each test. The 96-well plate was placed in a 37 ℃ incubator for 18 to 24 h, and the results were observed and recorded. The experiment was repeated three times.

### AMO and RSE combined drug sensitivity test

The methods of the combined drug sensitivity test of AMO and RSE were performed as previously reported in Fei Lin, et al. [42]. Based on the results of the single drug MIC tests, AMO (RSE) was diluted to 7 concentrations with broth, and 50 μL per well was added from the 7th horizontal row (vertical column) to the 1st horizontal row (vertical column) of 96-well plates from low concentration to high concentration. 100 μL of drug was added to the 8th vertical column (horizontal row), which was used as the single drug control of AMO (RSE). 100 μL of bacteria suspension was added in each well. 200 μL of bacterial suspension was added to the 11th vertical column as positive control wells. 200 μL of broth was added to the 12th vertical column as the negative control group. The 96-well plate was placed in a 37 ℃ constant temperature incubator for 18 to 24 h. The results were observed and recorded, and the test was repeated three times.

### Preparation of powder—compatibility testing of AMO-RSE and excipient screening

Nine samples of the same batch were randomly taken and divided into three groups. The first three samples were stored at 60 °C for 10 days. The second set of three samples was stored at a temperature of 25 °C and relative humidity of 90 ± 5% for 10 days, and the third set of three samples were subjected to illumination at 4500 lx ± 500 lx for 10 days. Samples were collected on days 5 and 10. According to the test results of key items of the API stability survey, the results were compared with the results on day 0.

For the screening of excipients, many excipients, such as glucose and sucrose, usually used for making powder or premix, were selected to investigate the quality of the powder, and the relative density, moisture absorption percentage, appearance uniformity, and mentioned bellows were the key indices to investigate the stability of the powder.

### Relative density test

The relative density of drugs and excipients was measured by the pycnometer method. A total of 1 g of the material to be tested was weighed, the weight was recorded as W1, and the sample was transferred to the pycnometer, which was filled with boiled cold water (temperature: 25 °C). The pycnometer was stoppered, and the weight was recorded as W2. The bottle was emptied and the contents washed, the bottle was filled with newly boiled cold water and weighed accurately, and the weight was recorded as W3. The relative density was calculated according to the following formula.

$$\boldsymbol R\boldsymbol e\boldsymbol l\boldsymbol a\boldsymbol t\boldsymbol i\boldsymbol v\boldsymbol e\boldsymbol\;\boldsymbol d\boldsymbol e\boldsymbol n\boldsymbol s\boldsymbol i\boldsymbol t\boldsymbol y\boldsymbol\;\boldsymbol o\boldsymbol f\boldsymbol\;\boldsymbol t\boldsymbol e\boldsymbol s\boldsymbol t\boldsymbol\;\boldsymbol s\boldsymbol a\boldsymbol m\boldsymbol p\boldsymbol l\boldsymbol e\boldsymbol\;\boldsymbol=\frac{{\mathbf W}_{\mathbf1}\boldsymbol\times\mathbf\rho}{{\mathbf W}_{\mathbf1}\boldsymbol-\boldsymbol({\mathbf W}_{\mathbf2}\boldsymbol-{\mathbf W}_{\mathbf3}\boldsymbol)}$$

Note: ρ represents freshly boiled cold water with a density of 0.99707 g/cm3 at 25 °C.

### Moisture absorption percentage test

Sample dried to a constant weight (1 g) was placed in a 5 mL flat weighing bottle, shaken gently to evenly distribute the sample, weighed accurately, put it in a dryer containing supersaturated sodium chloride solution (the weighing bottle is opened), and weighed after 24 h (*n* = 3). The moisture absorption percentage was calculated according to the following formula:

$$Moisture\;absorption\;percentage\;(\%)\;=\frac{\mathbf w\mathbf e\mathbf i\mathbf g\mathbf h\mathbf t\boldsymbol\;\mathbf a\mathbf f\mathbf t\mathbf e\mathbf r\boldsymbol\;\mathbf m\mathbf o\mathbf i\mathbf s\mathbf t\mathbf u\mathbf r\mathbf e\boldsymbol\;\mathbf a\mathbf b\mathbf s\mathbf o\mathbf r\mathbf p\mathbf t\mathbf i\mathbf o\mathbf n\boldsymbol-\boldsymbol\;\mathbf w\mathbf e\mathbf i\mathbf g\mathbf h\mathbf t\boldsymbol\;\mathbf b\mathbf e\mathbf f\mathbf o\mathbf r\mathbf e\boldsymbol\;\mathbf m\mathbf o\mathbf i\mathbf s\mathbf t\mathbf u\mathbf r\mathbf e\boldsymbol\;\mathbf a\mathbf b\mathbf s\mathbf o\mathbf r\mathbf p\mathbf t\mathbf i\mathbf o\mathbf n}{\mathbf w\mathbf e\mathbf i\mathbf g\mathbf h\mathbf t\boldsymbol\;\mathbf b\mathbf e\mathbf f\mathbf o\mathbf r\mathbf e\boldsymbol\;\mathbf m\mathbf o\mathbf i\mathbf s\mathbf t\mathbf u\mathbf r\mathbf e\boldsymbol\;\mathbf a\mathbf b\mathbf s\mathbf o\mathbf r\mathbf p\mathbf t\mathbf i\mathbf o\mathbf n}\boldsymbol\times\boldsymbol\;\mathbf{100}\boldsymbol\%$$

#### Angle of repose test

The fixed funnel method was used to measure the angle of repose. A funnel was fixed at a height of 3 cm above coordinate paper (the paper was placed on a horizontal table), and materials were added through the funnel until the top of the accumulated cone came into contact with the bottom of the funnel. The cone diameter was measured, and the angle of repose was calculated with the ratio of the distance between the funnel and paper to the cone radius as the tangent.

### Quality inspection of powder

#### Critical relative humidity test

Dry AMO-RSE powder was prepared according to the prescription ratio at a constant weight. Three compound powder samples, 1.0 g each, were put into flat weighing bottles with a constant weight, weighed accurately, and then placed without caps into a dryer with a different humidity. Moisture absorption occurred at a constant temperature until reaching a constant weight, each sample was accurately weighed, and the average moisture absorption percentage of particles under different relative humidity was calculated.

#### Appearance uniformity test

A small amount of test powder was put on smooth paper, spread to approximately 5 cm^2^, flattened surface, and observed in a bright place to evaluate whether it had uniform colour and no pattern or colour spots.

### Pharmacokinetics study

#### Animals

Twelve healthy Landrace × Large White hybrid pigs, half male and half female, with an average body weight of 15 ± 3.2 kg (range 11–18 kg), were randomly divided into two groups (three male and three female pigs in each group) for pharmacokinetics experiments and were purchased from JuCong agriculture, Nanning, Guangxi. Commercial feed and water free of antibiotics and target drugs were freely available to the pigs housed individually in 12 pig pens. After the experiment, the pigs were released.

The study was conducted according to the recommendations of the academy’s animal research guidelines and approved by the Animal Ethics Committee of Guangxi University (protocol number: GXU-2018–024).

All methods were carried out in accordance with relevant guidelines and regulations. All methods are reported in accordance with ARRIVE guidelines (https://arriveguidelines.org) for the reporting of animal experiments.

### Plasma sample preparation

Pig jugular vein blood was collected in an anticoagulant tube, and centrifuged at 3000 rpm for 10 min, and the separated plasma was stored at -20 ℃. The plasma sample was placed at room temperature for natural thawing, and it was mixed evenly with a vortex, 1 mL of the plasma sample was accurately measure and placed it in a 10 mL centrifuge tube with a cover. Then, 3 mL of 1% acetonitrile formate solution(20:80) was added, and the sample was swirled for 2 min and centrifuged at 14,000 rpm for 20 min. The liquid was then transferred to a clean 10 mL glass tube, and dried it with nitrogen in a 45 ℃ water bath. 1 mL of double distilled water was add for redissolution and the sample was swirled for 30 s, and passed through the column for standby. The HLB small column was activated with 5 mL of methanol and then balanced with 5 mL of water. The redissolved sample was passed through the column, washed with 2 mL of water, and eluted with 3 mL of acetonitrile. The eluate was collected, placed in a water bath at 45 °C, and dried with nitrogen. Add 250 μL of double distilled water was added for redissolution, 0.22 μM of water was added of system membrane filtration, and the volume of the sample injection was 100 μL.

### Analysis of plasma AMO content

Using HPLC to measured the content of AMO. The chromatographic conditions were as follows: the column was an Phenomenex luna C18; detection wavelength: 254 nm; column temperature: 40 °C; mobile phase: 0.2% formic acid water and acetonitrile with the ratio of 1000:20; flow rate: 1.00 mL/min; and injection volume: 100 µL. The plasma concentration of amoxicillin had a good linear relationship in the range of 0.1–10 µg/ml, and the correlation coefficient was 0.9993. The limits of detection and quantification of amoxicillin in the plasma were 0.03 and 0.1 µg/ml, respectively.

### Analysis of plasma BCL content

The content of BCL were measured using HPLC. The chromatographic conditions were as follows: the column was an Phenomenex luna C18; detection wavelength: 278 nm; column temperature: 40 °C; mobile phase: 0.4% acetic acid water and methyl alcohol with the ratio of 50:50; flow rate: 1.00 mL/min; and injection volume: 50 µL. The plasma concentration of BCL had a good linear relationship in the range of 0.1–10 µg/ml, and the correlation coefficient was 0.9981. The limits of detection and quantification of amoxicillin in the plasma were 0.03 and 0.1 µg/ml, respectively.

### Experimental design for the initial pharmacokinetic study

In the first study, the pharmacokinetics of AMO and BCL in the compound formulation were compared with formulations containing the same active ingredients and content with only AMO or BCL. A crossover study design was conducted to randomize assignment to one of the three formulations. The washout period was two weeks between treatment administrations. Each pig was subjected to the following three treatments via oral administration: (a) AMO-RSE powder (b) 10 mg/kg AMO and (c) 5 mg/kg RSE. At predetermined time points (0 min, 10 min, 20 min, 30 min, 45 min, 1 h, 1.5 h, 2 h, 3 h, 4 h, 6 h, 8 h, 12 h, 24 h), 5 ~ 8 mL of blood sampling from jugular vein 5–8 mL, put into heparinized centrifuge tubes, and centrifuged at 3000 rpm for 10 min. The upper plasma was absorbed, subloaded into 2 mL EP tubes, and stored in a -20 °C freezer for subsequent HPLC analysis.

Equation for the area under the curve using the linear- log Trapezoidal rule:

$${\varvec{A}}{\varvec{U}}{{\varvec{C}}}_{0-\boldsymbol{\infty }}={\varvec{A}}{\varvec{U}}{{\varvec{C}}}_{0-{\varvec{l}}{\varvec{a}}{\varvec{s}}{\varvec{t}}}+\frac{{{\varvec{C}}}_{{\varvec{t}}}}{{{\varvec{\lambda}}}_{{\varvec{z}}}}$$

AUC_0-last_ is the area under the drug-time curve from time 0 to the time (last) corresponding to the last time the blood drug concentration can be measured, and C_t_ is the blood drug concentration measured at the last time point in the experiment, λ_Z_ is the elimination rate constant in the single-exponential decay equation fitted to the end of the curve.

### Experimental design for the second pharmacokinetic study

Another set of 6 pigs, different from those in the initial pharmacokinetic study, were used to conduct comparative pharmacokinetics. A crossover study design was implemented to three different doses of AMO-RSE powder by oral administration, namely, AMO was administered at a dose of 10, 20 and 30 mg/kg, and the dose of BCL was 5, 10, 15 mg/kg. The time points for plasma collection were the same as those in the first study.

### Statistical analysis

The concentrations of AMO and BCL in plasma at each time point are expressed as the arithmetic mean ± SD. The pharmacokinetic parameters were calculated by using noncompartmental analysis based on statistical moment theory. The software SPSS 10.0 (SPSS Inc, Chicago, IL, USA) and Window's Microsoft (R) Excel were used to complete the calculation and analysis of the data, and the software SigmaPlot 13.0 and OrignPro 9.1 were used to calculate the regression equation and relevant statistical parameters, as well as for graph generation.

## Data Availability

The datasets used and analyzed during the current study are available from the corresponding author upon reasonable request.

## Abbreviations

AMO: Amoxicillin
RSE: Radix Scutellariae extract
Cmax: Maximum plasma concentration
Tmax: Time to Cmax
AUC: Area under the concentration–time curve
T ½ λ: Terminal half-life
λ: Terminal rate constant
MRT: Mean residence time extrapolated to infinity
*E. coli*: *Escherichia coli*
*S. aureus*: *Staphylococcus aureus*
MIC: Minimum inhibitory concentrations
PK: Pharmacokinetics
API: Active pharmaceutical ingredient
p.o: Oral administration
PK/PD: Pharmacokinetics/pharmacodynamics
